# Supplementary material for: Content-rich biological network constructed by mining PubMed abstracts
Source: BMC Bioinformatics. 2004 Oct 8;5:147. doi: 10.1186/1471-2105-5-147 (PMC528731; doi:10.1186/1471-2105-5-147)
Supplement: Additional File 2 — The original results of the above study (non-essential files are deleted to keep the file size under the limit set by BMC bioinformatics). [file 1471-2105-5-147-S2.bz2 › chilibotAdditionalFile2/dip05/437ID9430724E10382/html/CCL17_CCR4.html]

 


 **CCL17** and **CCR4** 
  
Found 87 abstracts in PubMed, retrieved 05.  
 

 What does Google say? 
 PDF only 
| .edu only 

---

**Interactive relationship** (e.g. stimulation, inhibition, etc)

**Neutral relationship**- TARC  [ **CCL17** ]  interacts primarily with the  **CCR4**  receptor and to a lesser extent with the CCR8 receptor.  Ref: 12832759 Acta Crystallogr D Biol Crystallogr, 2003
- Since the ligand of the TARC  [ **CCL17** ]  and MDC is  **CCR4** , which is specifically expressed on Th2 type T cells, the production of these CC chemokines is likely to play important roles in the T cell and B cell interaction.  Ref: 12715916 J Med Dent Sci, 2003
- CC chemokine receptor 4  [ **CCR4** ]   **CCR4**  has been identified as a specific receptor for both thymus and activation regulated chemokine  [ **CCL17** ]  TARC  [ **CCL17** ]  and macrophage derived chemokine MDC, and is preferentially expressed on Th2 cells.  Ref: 12757454 Allergy, 2003
- In addition,  **CCR4** , which is characteristic Th2 helper phenotype, and its ligand thymus and activation regulated chemokine  [ **CCL17** ]  TARC  [ **CCL17** ] , were compared with CXCR3, as Th1 phenotype.  Ref: 12688353 Leuk Lymphoma, 2003
- Three chemokines in particular, CXCL12 SDF 1,  **CCL17**  TARC  [ **CCL17** ] , and CCL22 MDC, recently have been shown to be strong and rapid activators of platelet aggregation and adhesion after their binding to platelet CXCR4 or  **CCR4** , when acting in combination with low levels of primary agonists.  Ref: 12851650 Microcirculation, 2003

**Non-interactive relationship** (e.g. studied together, co-existance, homology, etc.)

- TARC  [ **CCL17** ]  positive cells and  **CCR4**  positive cells were rarely encountered in all cases.  Ref: 12688353 Leuk Lymphoma, 2003
- Accumulation of  **CCR4**  expressing CD4 T cells and high concentration of its ligands TARC  [ **CCL17** ]  and MDC in bronchoalveolar lavage fluid of patients with eosinophilic pneumonia.  Ref: 12757454 Allergy, 2003
- CONCLUSIONS Our data suggest that Th2 cells, which express  **CCR4**  and its ligands TARC  [ **CCL17** ]  and MDC, contribute to the pathogenesis of EP in the lung.  Ref: 12757454 Allergy, 2003
- There was a significant correlation between the number of  **CCR4**  CD4 T cells and the levels of TARC  [ **CCL17** ] , MDC, and IL 5 in BALF of patients with EP.  Ref: 12757454 Allergy, 2003
